# From serological surveillance to the identification of native human cases of hantavirus pulmonary syndrome in French Guiana

**DOI:** 10.1186/1753-6561-5-S1-P54

**Published:** 2011-01-10

**Authors:** S Matheus, F Djossou, D Moua, D Hommel, P Dussart, B de Thoisy, V Lacoste, A Lavergne

**Affiliations:** 1 Laboratoire de virologie, Centre National de Référence des Arbovirus, Institut Pasteur de la Guyane, Cayenne, French Guiana; 2 Centre Hospitalier Andrée Rosemon, Cayenne, French Guyana; 3 Laboratoire des Interactions Virus-Hôtes, Institut Pasteur de la Guyane, Cayenne, French Guyana

## 

Hantaviruses are rodent-borne negative-sense RNA viruses belonging to the *Bunyaviridae* family, genus Hantavirus. These emerging viruses cause cardiopulmonary syndrome in North and South America, which is a respiratory illness following the inhalation of dust contaminated by infectious rodent feces or urine.

Until recently, no information was available related to the presence of hantavirus in French Guyana, a French department in South America. Nevertheless, the description of atypical pneumonia cases unrelated to any known etiological agent and the identification of hantavirus reservoirs in neighboring countries led us to conduct a serological study in a collection of sera from patients who had presented compatible symptoms: the prevalence of IgG antibodies to hantavirus in this population was 1.42%.

After those retroactive results, systematic hantavirus serology screening was implemented in every newcoming patient with suggestive etiology. This led us to identify a native case in French Guyana. After this first case, a second case was registered 1 year later in December 2009 (in a periurban area). Molecular analyses were conducted to characterize genetically these two strains of hantavirus. Complete sequences of the S segment were obtained and phylogenetic analyses confirmed that strains isolated in French Guyana and tentatively named *Maripa* virus belong to the Rio Mamore species.

Human hantavirus epidemics are associated with fluctuations of rodent populations, caused by climatic, ecological and environmental changes, or growing human activities associated with nature or agriculture. In Guyana, 90% of the land is still tropical rain forest, but economic development results in growing pressures on natural habitats. Continuous surveillance of the virus in the human population would be beneficial. Furthermore, surveys of potential reservoirs may help to understand hantavirus dispersion and to reduce the risk of viral emergence.

